# PTEN mediates the cross talk between breast and glial cells in brain metastases leading to rapid disease progression

**DOI:** 10.18632/oncotarget.14047

**Published:** 2016-12-20

**Authors:** Ina Hohensee, Han-Ning Chuang, Astrid Grottke, Stefan Werner, Alexander Schulte, Stefan Horn, Katrin Lamszus, Kai Bartkowiak, Isabell Witzel, Manfred Westphal, Jakob Matschke, Markus Glatzel, Manfred Jücker, Tobias Pukrop, Klaus Pantel, Harriet Wikman

**Affiliations:** ^1^ Institute of Tumor Biology, University Medical Center Hamburg-Eppendorf, Hamburg, 20246, Germany; ^2^ Department of Hematology/Oncology, University Medical Center Göttingen, 37099 Göttingen, Germany; ^3^ Institute of Biochemistry and Signal Transduction, University Medical Center Hamburg-Eppendorf, 20246 Hamburg, Germany; ^4^ Department of Neurosurgery, University Medical Center Hamburg-Eppendorf, 20246 Hamburg, Germany; ^5^ Bone Marrow Transplantation Unit, University Medical Center Hamburg-Eppendorf, 20246 Hamburg, Germany; ^6^ Department of Gynecology, University Medical Center Hamburg-Eppendorf, 20246 Hamburg, Germany; ^7^ Institute of Neuropathology, University Medical Center Hamburg-Eppendorf, Hamburg-Eppendorf, 20246 Hamburg, Germany; ^8^ Department of Medicine III, University Medical Center Regensburg, 93053 Regensburg, Germany

**Keywords:** brain metastases, breast cancer, PTEN, microenvironment, astrocytes

## Abstract

Despite improvement of therapeutic treatments for breast cancer, the development of brain metastases has become a major limitation to life expectancy for many patients. Brain metastases show very commonly alterations in EGFR and HER2 driven pathways, of which PTEN is an important regulator. Here, we analyzed PTEN expression in 111 tissue samples of breast cancer brain metastases (BCBM). Loss of PTEN was found in a substantial proportion of BCBM samples (48.6%) and was significantly associated with triple-negative breast cancer (67.5%, *p* = 0.001) and a shorter survival time after surgical resection of brain metastases (*p* = 0.048). Overexpression of PTEN in brain-seeking MDA-MB-231 BR cells *in vitro* reduced activation of the AKT pathway, notably by suppression of Akt1 kinase activity. Furthermore, the migration of MDA-MB-231 BR cells *in vitro* was promoted by co-culturing with both astrocytes and microglial cells. Interestingly, when PTEN was overexpressed the migration was significantly inhibited. Moreover, in an *ex vivo* organotypic brain slice model, PTEN overexpression reduced invasion of tumor cells. This was accompanied by reduced astrocyte activation that was mediated by autocrine and paracrine activation of GM-CSF/ CSF2RA and AKT/ PTEN pathways. In conclusion, loss of PTEN is frequently detected in triple-negative BCBM patients and associated with poor prognosis. The findings of our functional studies suggest that PTEN loss promotes a feedback loop between tumor cells and glial cells, which might contribute to disease progression.

## INTRODUCTION

The molecular basis of organ-specific metastasis patterns is currently under intensive investigation but remains unclear for most parts. The so called ‘seed-and-soil’ theory, introduced in 1889 by Paget, proposed that metastatic tumor cells (“seed”) need to harbor specific properties that enable them to interact with the microenvironment at the metastatic site (“soil”) allowing their homing and outgrowth to metastases [1]. Brain parenchyma offers particular conditions to metastasizing tumors cells due to both the blood-brain barrier and its extremely specialized microenvironment. Importantly, this microenvironment impedes tumor cell treatment as the impermeability of the blood-brain barrier prevents many drugs from entering the brain parenchyma and other drugs are not well tolerated by the brain. Therefore, the prognosis of cancer patients with brain metastases is often extremely poor and there is an urgent clinical need to deepen our understanding of factors leading to brain metastasis formation.

There are different types of glial cells in the brain, of which mainly two have been associated with brain metastases, i.e. astrocytes and microglia [2]. Both astrocytes and microglia are non-proliferative in normal adult brain, but upon injury can be rapidly activated. Active astrocytes express glial fibrillary acidic protein (GFAP) and show conformational changes that can lead to glial scar formation or gliosis [3]. In many neuroinflammatory diseases such as Alzheimer`s and Parkinson`s disease astrocytes have both an important neurotoxic and neuroprotective role [2]. A similar role has been suggested for the interaction of astrocytes with tumor cells [2, 4–6]. Both microglia and astrocytes are known to secrete a multitude of chemokines upon contact with tumor cells and in experimental settings they have been shown to enhance the colonization of tumor cells [7, 8]. Furthermore, in clinical samples it has been shown that both astrocytes and microglia may accumulate outside and inside the metastases with an impact on patient outcome [9].

Breast cancer is a heterogeneous tumor entity categorized into different molecular subtypes characterized by variable disease progression, therapy response and organotropism [8]. Luminal tumors are correlated with positive hormone receptor status, a relatively good prognosis and mainly bone metastases. HER2-positive tumors harbor a high-level amplification of the *HER2* gene, whereas the triple-negative or basal-like subtype is associated with hormone receptor- and HER2-negative status. Moreover, HER2-positive and triple-negative tumors possess a higher risk of metastasizing to the brain compared to luminal tumors [10]. Among triple-negative breast tumors, brain metastases may occur early and more frequently as the first site of relapse compared to the other subtypes. Additionally, triple-negative brain metastasis patients have the worst prognosis among breast cancer subtypes, partially due to the absence of a distinct molecular characterization that would facilitate the use of targeted therapies. In general, the prognosis of brain metastases is extremely poor; if left untreated the median survival is only 1–2 months [8, 11]. Therefore, the development of improved management strategies for BCBM is an important clinical challenge.

We and others have shown the important role of EGFR and HER2 signaling in breast cancer brain metastasis (BCBM) formation [12, 13]. Alterations in both epidermal growth factor receptor (EGFR) and/or phosphatase and tensin homologue (PTEN) are associated with the triple-negative subtype [12, 14]. Interestingly, highly aggressive primary brain tumor glioblastomas are characterized by frequent EGFR and PTEN alterations [15]. These findings suggest a role for EGFR/PTEN alterations in driving cerebral colonization. In this study, we aimed to elucidate the clinical and functional role of PTEN specifically in BCBM. For this purpose, we first assessed the clinical relevance of PTEN expression in a large cohort of BCBM samples. Furthermore, we overexpressed PTEN in the brain-seeking basal breast cancer cell line MDA-MB-231 BR and analyzed its effect in glial cell microenvironment.

## RESULTS

### Evaluation of PTEN expression and clinical relevance in BCBM samples

**Figure 1:**
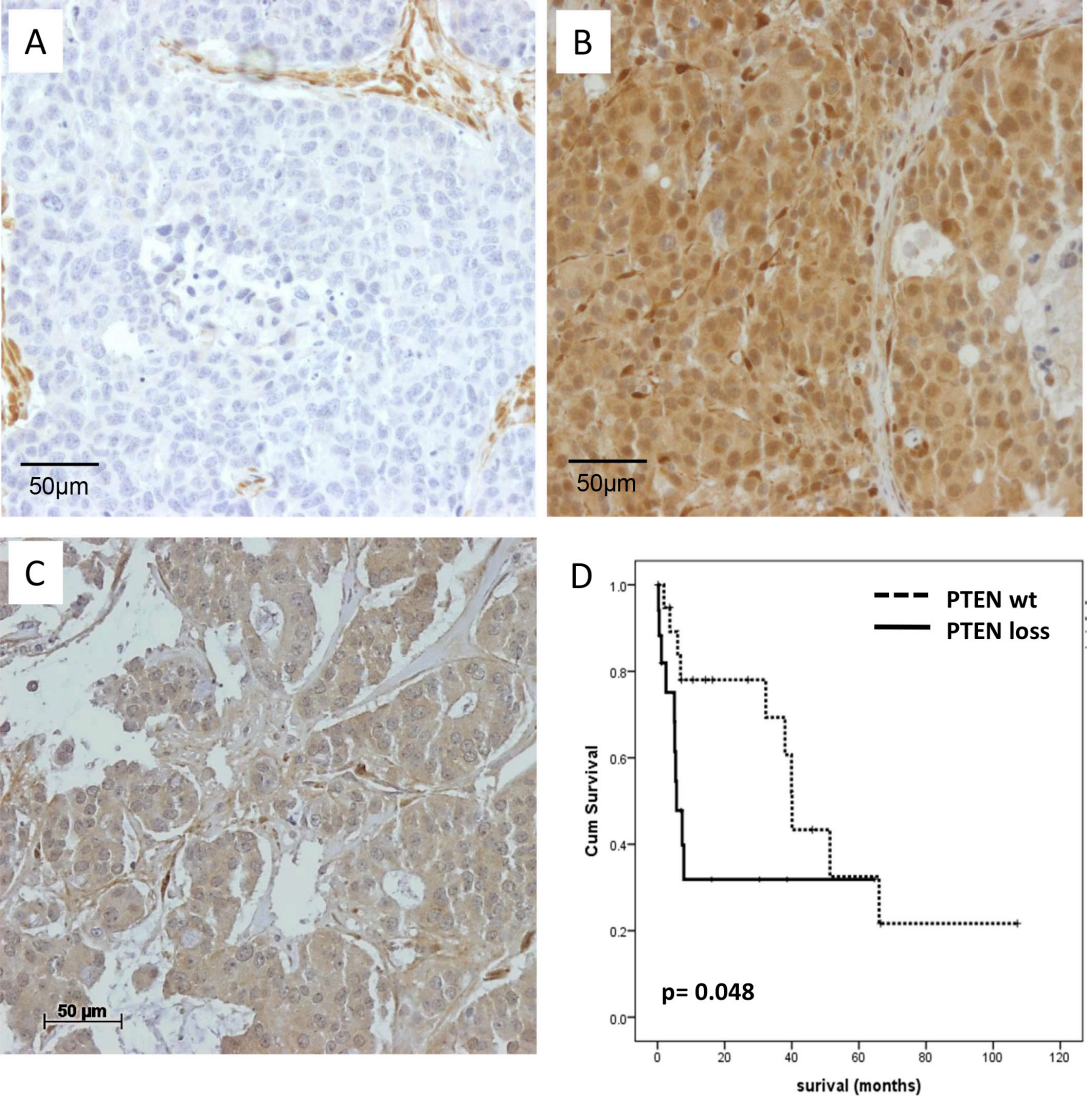
PTEN protein expression in BCBM samples (**A**) PTEN negative BCBM sample with PTEN positive stromal cells (**B**) Strong positive PTEN immunohistochemical staining results of a BCBM sample in both the cytoplasm and tumor nucleus (**C**) Positive PTEN staining of the cytoplasm. (**D**) Kaplan–Meier survival analysis of PTEN expression in BCBM samples. Survival differences were analyzed by log rank test.

PTEN protein expression was assessed by immunohistochemistry in 111 BCBM cases out of 131 samples placed on the TMA (Figure 1A, 1B). Of these samples, 48.6% were classified as PTEN negative (Table 1). Loss of PTEN expression was significantly associated with hormone receptor negative (57.6%; *p* = 0.001) and HER2 negative (83.7%; *p* = 0.003) BCBM status. When these samples were classified into molecular subtypes, 67.5% of all triple-negative brain metastases samples were negative for PTEN, whereas only 29.3% of HER2 positive and 30.0% of hormone receptor positive samples were negative for PTEN expression (*p* = 0.01). Kaplan Meyer analysis identified loss of PTEN to be significantly associated with a shorter survival time after brain metastases resection (*p* = 0.048, Figure 1C).

**Table 1:** PTEN protein expression in brain metastases

	PTEN pos.		PTEN neg.		*p*-value
	*n*	%	*n*	%	
**All**	**57**	*51.4*	**54**	*48.6*	
**Age at brain OP**					
< 50 years	29	*49.2*	30	*50.8*	0.705
> 50 years	28	*53.8*	24	*46.2*	
Hormone receptor*					
positive	26	*76.5*	8	*23.5*	**0.001**
negative	28	*42.4*	38	*57.6*	
HER2^*^*					
negative	8	*16.3*	41	*83.7*	**0.003**
positive	29	*70.7*	12	*29.3*	
Subtype^**^*					
HR positive	14	*70.0*	6	*30.0*	**0.001**
HER2 positive	29	*70.7*	12	*29.3*	
TNBC	13	*32.5*	27	*67.5*	

*information missing for 11 cases, **information for 21 cases; *** information missing for 30 cases.

### Establishment of a PTEN inducible cell line and measurements of Akt activation

**Figure 2:**
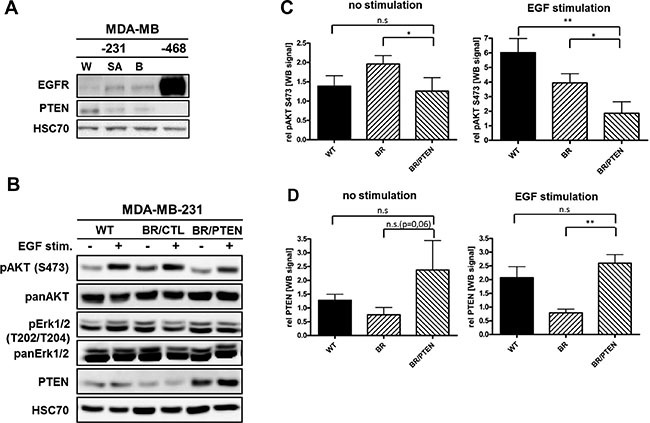
EGFR and PTEN expression in basal breast cancer cell lines (**A**) Immunoblot analysis of EGFR and PTEN in parental MDA-MB-231 (WT), its bone- (SA) as well as brain-seeking (BR) sublines and MDA-MB-468 (EGFR-amplified) cells. Protein levels of whole cell lysates were visualized by EGFR- and PTEN-specific antibodies. HSC-70 served as loading control (**B**) Immunoblot analysis of different PTEN pathway members in parental MDA-MB-231 (WT) cell line, brain-seeking (BR) sublines with either empty vector control (BR/CTL) or PTEN overexpression (BR/PTEN). Protein levels of whole cell lysates treated with 20 nM EGF (+) were compared to untreated cells (-) and visualized by antibodies specific for phosphorylated AKT (pAKT S473), total AKT (panAKT), phosphorylated Erk1/2 (pErk1/2 T202/T204), and total Erk1/2 (panErk1/2) and PTEN. HSC70 served as loading control. (**C**) Quantification of the pAKT S473 and (**D**) PTEN expression (both triplicate experiments) before and after EGF treatment. Samples were normalized to the corresponding HSC70 expression (Bars: SD. n.s.: not significant, *p < 0,05).

EGFR and PTEN protein levels were analyzed in different subclones of MDA-MB-231 triple-negative breast cancer cell lines by immunoblotting (Figure 2A). Parental MDA-MB-231 (WT) cells expressed the highest amounts of PTEN and the lowest amounts of EGFR. Bone- (SA) and brain-seeking (BR) lines showed reduced PTEN protein expression compared to the parental cell line, whereas EGFR protein levels showed an inverse expression pattern. Differential expression in the metastatic sublines compared to parental MDA-MB-231 cells suggest a potential relevance of PTEN loss and EGFR overexpression in breast cancer metastasis. MDA-MB-231 BR cells were selected to analyze effects of PTEN overexpression on cellular processes in context of brain microenvironment. To study effects of elevated PTEN expression, MDA-MB-231 BR cells were transduced by lentiviral particles containing either the PTEN coding sequence (pPTENiZs2puro^++^tTR^+^) under control of doxycycline response element (231BR/PTEN) or empty vector (piZs2puro^++^tTR^+^) (231BR/CTL).

To validate PTEN function in the established cell lines, cells were treated with the EGFR ligand, EGF, known to induce AKT phosphorylation at Serine 473 (S473) [16]. Following stimulation, AKT activation was detected in parental (WT) and control (231BR/CTL) cells but activation was significantly diminished in PTEN overexpressing MDA-MB-231-BR (231BR/PTEN) cells (Figure 2B). Accordingly, PTEN overexpression in MDA-MB-231 BR cells lead to reduced EGFR/HER2 pathway activation as indicated by diminished phosphorylation of AKT proteins. Conversely, no effect on ERK/MAPK signaling could be detected by either EGF treatment or PTEN overexpression in this cell line (Figures 2B and Supplementary Figure S1A).

**Figure 3:**
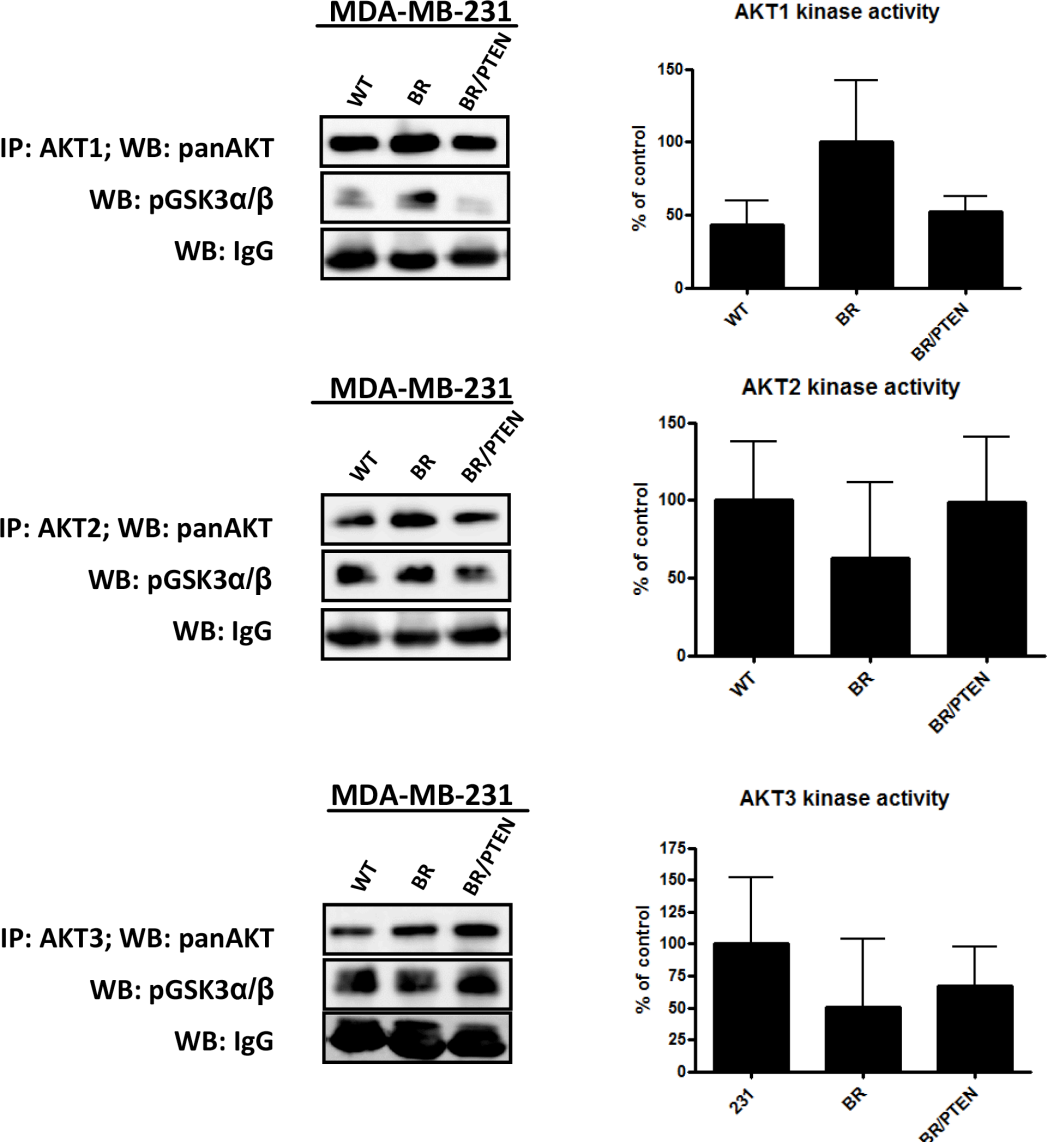
AKT isoform specific in vitro kinase assays AKT isoform specific kinase activity in parental MDA-MB-231 (WT) cells, its brain seeking (BR) subline cells and PTEN overexpressing MDA-MB-231 BR cells (BR/PTEN) was analyzed by immunoprecipitation of AKT isoforms followed by incubation with AKT substrate GSK3α/β fusion protein. Phosphorylation at S9/21 was detected by Western blot analysis. Relative AKT isoform activity was quantified from Western blot analysis of triplicates, normalized to the corresponding mouse IgG level for sample correction.

To assess whether a particular AKT isoform is activated in the brain seeking MDA-MB-231 BR subline and is repressed when PTEN is overexpressed, *in vitro* kinase assays specific for individual AKT isoforms were performed. Both cell lines express all three AKT isoforms i.e. Akt1, Akt2 and Akt3 (Figure 3). Measurement of the AKT isoform activities revealed that, Akt1 activity, but not Akt2 or Akt3 activity, was increased in the subline (Figure 3). Expression of PTEN in the MDAMB-231BR subline reduced the activity of Akt1 to the level observed in the less aggressive MDA-MB-231 WT cells (Figure 3). These data indicate that the observed activation of AKT in the brain seeking subline is due to an isoform specific activation of the Akt1 isoform and that this activation can be reversed by expression of PTEN in these cells.

### Effects of glial cells on tumor cell proliferation *in vitro*

Previously, we demonstrated that tumor cells influence the proliferation of macrophages and can partially rescue the inhibition of essential growth factors [16] as well as microglia and astrocytes could enhance the aggressiveness of tumor cells via secreted factors and vesicles [4, 17]. Regarding the previous observations, we analyzed the influence of PTEN in this signaling loop. Since PTEN dependent signaling is known to regulate cellular processes like tumor cell growth, we analyzed the proliferation of 231BR/CTL and 231BR/PTEN in the context of brain microenvironment. Therefore, we treated 231BR/CTL and 231BR/PTEN cells with conditioned media (CM) from activated astro- or microglia cultures. CM was produced through activating astrocytes either by co-culturing them in the presence of the tumor cells (CM: A 231BR) or by treatment with IFNγ and TNFα (CM: A IFNγ + TNFα), two agents known to activate astrocytes [18].

**Figure 4:**
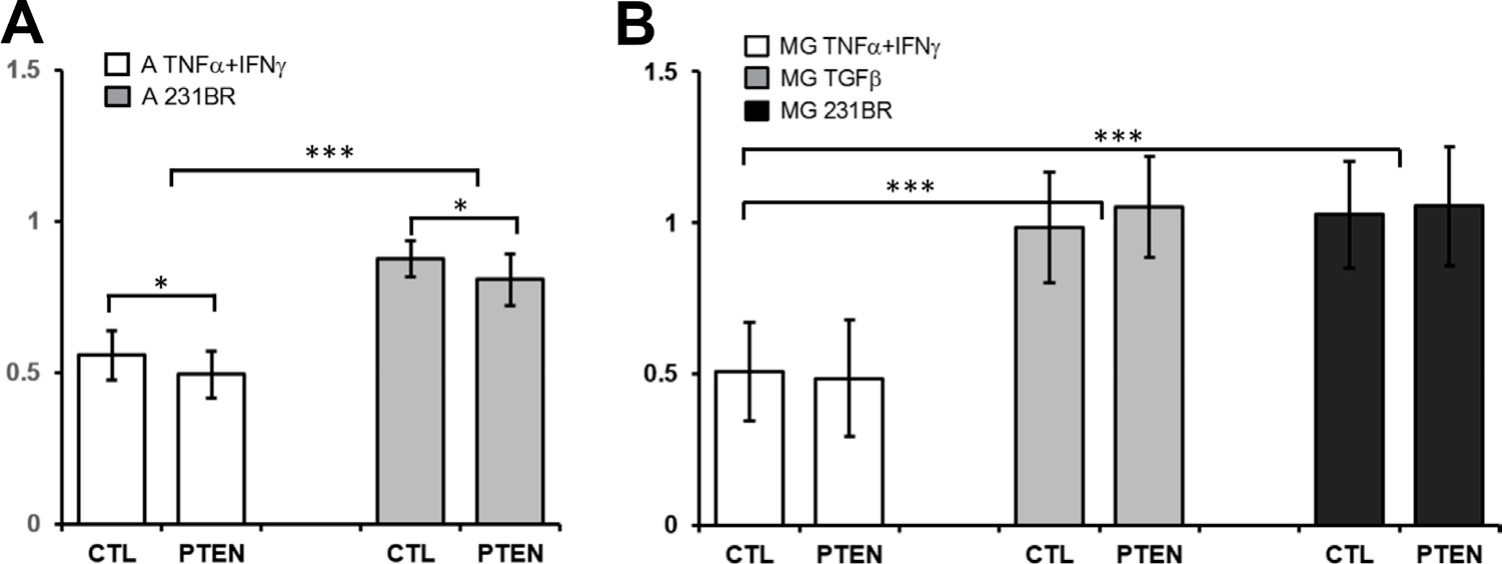
Effects of glial cell conditioned media on MDA-MB-231 BR cell proliferation (**A**) Control (CTL) and PTEN overexpressing MDA-MB-231 BR cells (PTEN) were cultured for 96 h in 96-well plates in conditioned media of astrocytes treated with 20 nM TNFα and 20 nM IFNγ (A TNFα + IFNγ) or in media from astrocytes cocultured with tumor cells (A 231BR). Proliferation was estimated using MTT assay approach and a ratio was calculated against tumor cells grown in conditioned media of astrocyte single cultures. (**B**) Same procedure was used to determine proliferation rate of CTL and PTEN cells cultured in conditioned media of microglia treated with 20 nmol TNFα and 20 nmol IFNγ (MG TNFα + IFNγ), 20 nmol TGFβ1 (MG TGFβ1) or cocultured with tumor cells (A 231BR) against conditioned media of microglia single coculture. *p < 0.05, ***p < 0.001.

MTT assays revealed a significantly diminished proliferation of both 231BR/CTL and 231BR/PTEN (ratio 0.56 and 0.50) cells when cultured in CM from IFNγ- and TNFα-activated astrocytes compared to CM from astrocytes cultured in standard media (*p* < 0.05; Figure 4A). In contrast, no anti-proliferative effects could be detected in cultures with CM from co-cultured astrocytes and tumor cells (ratio 231BR/CTL: 0.88, 231BR/PTEN: 0.81). Thus, proliferation of tumor cells independent of PTEN status was significantly reduced in the presence of IFNγ- and TNFα-activated astrocyte-CM compared to normal culture media or to CM from co-cultures of astrocytes with tumor cells (231BR/CTL and 231BR/PTEN: *p* < 0.001). In both settings PTEN overexpression caused a small but significant reduction of proliferation (Figure 4A, p < 0.05).

CM from microglia was produced similarly by co-culturing microglial cells with 231BR/CTL or 231BR/PTEN cells (CM: 231BR) or by treating them with IFNγ and TNFα (CM: MG IFNγ + TNFα) or TGFβ1 for 72 h (CM: MG IFNγ + TNFα). Interestingly, proliferation of both tumor cell lines was significantly reduced when cultured in CM of IFNγ- and TNFα-activated microglia (*p* < 0.001, ratios 0.51 and 0.49), but not by TGFβ1-treated microglial CM (Figure 4B, ratios 0.98 and 1.05). Similarly to astrocytes, no such effect could be detected in cultures with CM from co-cultured microglia and tumor cells (ratio 1.03 and 1.06). PTEN expression had no effect on the proliferation of tumor cells in these coculture settings.

### Effects of glial cells on tumor cell migration *in vitro*

**Figure 5:**
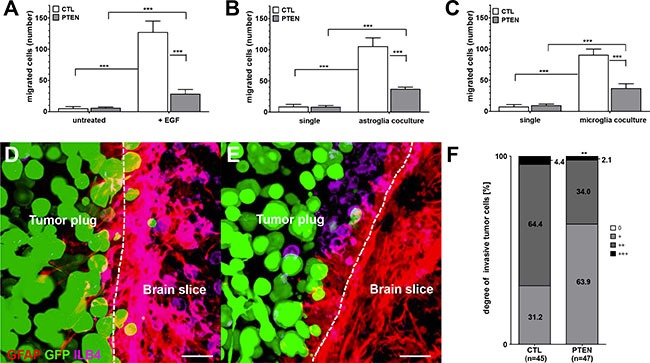
Effects of PTEN overexpression on migratory and invasion capacity (**A**) Migration of control (CTL) and PTEN overexpressing MDA-MB-231 BR cells (PTEN) seeded in transwell inserts was evaluated in 24-well plates using standard DMEM compared to DMEM supplemented with 20 nM EGF as chemoattractant. Additionally, astro- (**B**) or microglia (**C**) were used as chemoattractant and migration was compared against DMEM (untreated). Migrated cells on the bottom site of the insert were counted after 20 h incubation. Invasion was determined in organotypic brain slice cocultures of 6–8 day old mice and ZsGreen-positive (green) control (**D**) or PTEN overexpressing MDA-MB-231 BR cells (**E**) Cocultures were incubated for 96 h and double-stained against GFAP (astrocytes in red) as well as ILB4 (microglia in purple). The contact section between brain slice and tumor spheroid is visualized as a broken line. Scale bars represent 50 μm (**E**, **F**) The degree of tumor invasion was based on the following scoring system (0 = no cells; + <1/3; ++ = 1/3 - 2/3; +++ ≥ 2/3 invaded cells) relating to the fraction of contact section being invaded (**F**) Invasion was calculated of control (CTL) and PTEN overexpressing MDA-MB-231 BR cells (PTEN) in 45 and 47 brain slices. Student's *t-test* was used to analyze statistical significance. **p < 0.01, ***p < 0.001.

To assess whether PTEN expression is connected with migratory behavior of breast tumor cells, Boyden chamber assays with 231BR/CTL and 231BR/PTEN cells were performed by using EGF-supplemented DMEM or DMEM in the presence of astrocyte or microglia cells as chemoattractants. Astrocytes or microglia were seeded in 24-well plates 72 h prior to seeding 231BR/CTL or 231BR/PTEN cells into transwell inserts. Migration rate was determined by counting migrated cells from the lower part of the insert 20 h after seeding of tumor cells. The Boyden chamber assay revealed that tumor cells do not migrate without a chemoattractant but can be highly mobilized by EGF (Figure 5A). Furthermore, PTEN overexpression reduced significantly the migratory capacity (Figure 5A, all *p* < 0.001). Indirect co-cultures of both astrocytes as well as microglia also induced tumor cell migration comparable to EGF-treated media. Furthermore, once again migratory capacity of MDA-MB-231 BR cells was shown to be dependent on PTEN expression under both conditions, i.e. using either microglia or astrocytes as chemoattractants (Figure 5B and 5C, all *p* < 0.001).

### Effects of PTEN overexpression on tumor cell brain invasion

To get further insight into the dynamic interactions between PTEN overexpressing tumor cells and brain microenvironment, we performed *ex vivo* organotypic brain slice co-culture experiments [19]. In this setting, fresh brain sections of 6–8 day old mice were co-cultured with a spheroid of extracellular matrix embedded ZsGreen-expressing tumors cells for 96 h. Invasion was estimated in 45 and 47 brain sections for both 231BR/CTL and 231BR/PTEN cells, respectively. Astrocytes were stained with GFAP and visualized with TRITC antibodies. Visualization of microglia was performed by fluorescence dye-conjugated ILB4-Alexa Fluor 647.

**Figure 6:**
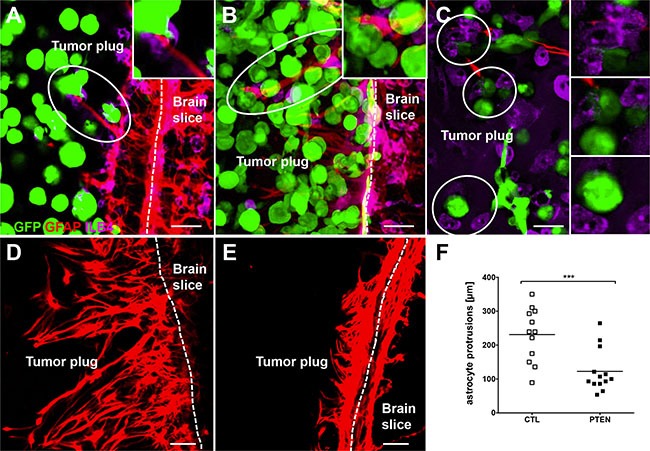
Interaction of tumor cells with brain microenvironment Control (**A**) as well as PTEN overexpressing (**B**) MDA-MB-231 BR cells interacted directly with both astro- (A and B) and microglia (**C**) in organotypic brain slice cocultures. Astrocyte built significantly longer protrusions in control cells culture (**D**) compared to coculture with PTEN overexpressing MDA-MB-231 BR cells (**E**) MDA-MB-231 BR cells expressed Zsgreen (green), astrocytes were stained against GFAP (red) and microglia very visualized by ILB4 staining (purple). Scale bars represent 50 μm. Kruskal-Wallis test was used to analyze statistical significance (**F**) ***p < 0.001.

Evaluation of the organotypic brain slice co-cultures revealed that 231BR/PTEN cells possess a significantly reduced invasion capacity compared with 231BR/CTL cells (*p* < 0.001) (Figure 5D–5E). Furthermore, both astrocytes as well as microglia interacted directly with the tumor cells (Figure 6A–6C). As previously demonstrated, microglial cells enter the 3D-tumor cell spheroid and interact with the tumor cells with different consequences [4, 7]. For example, inhibition of CXCR4 reduced the microglial chemotaxis towards the tumor cells. However, the PTEN modification of the tumor cells demonstrated no influence on the chemotaxis and interaction with the tumor cells with the microglia (data not shown). In contrast to microglia, the PTEN modification influenced the interaction between the astrocytes and tumor cells. We also previously demonstrated that astrocytes interact with tumor cells via long protrusions in the 3D tumor plug. We indicated this interaction as a criterium for activating astrocytes against tumor cells [4]. Interestingly, the length of astrocytes protrusions was significantly decreased in 231BR/PTEN co-cultures compared to co-cultures with 231BR/CTL cells (Figure 6D–6FD, p < 0,001), indicating that the 231BR/PTEN tumor cells have a reduced activation status compared to 231BR/CTL. A recent study on glioblastomas indicated that many glioma cells extend ultra-long membrane protrusions, and use these microtubes as routes for brain invasion [20].

The neuronal growth-associated protein 43 (Gap43) was shown to be a key component of this microtube formation and tumour cell invasion and proliferation [20]. We also investigated the Gap43 expression in both the tumor cells and astrocytes when either grown alone or in co-culture. Interestingly, the tumor cells did not express Gap43 when cultured alone but the expression was highly induced by astrocytes. In astrocytes basal levels of Gap43 expression were slightly elevated when grown in the co-culture setting. PTEN did not, however, seem to play a role in this activation process (Supplementary Figure S1B)

### Effect of PTEN expression on cytokine expression and secretion

**Figure 7:**
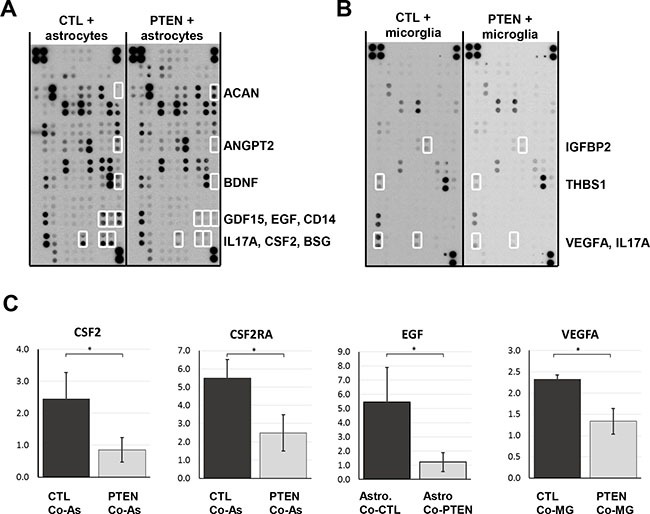
Effects of glial cell cocultures on cytokine secretion Control and PTEN overexpressing MDA-MB-231 BR cells were seeded in transwell inserts and cocultured for 48 h with astrocytes (**A**) or microglia (**B**). Antibody-spotted membranes were incubated with conditioned media of these cultures and levels of secreted proteins were visualized by HRP-conjugated antibodies. Proteins with a more than 2-fold expression difference are shown by open boxes. Results were verified by qPCR analyses (**C**). Astrocyte coculture reduced EGF and GM-CSF expression significantly in PTEN overexpressing (PTEN CoA) compared to control cells (CTL CoA, C). Astrocytes express the GM-CSF receptor (CSFR2A) only when cocultured with tumor cells. Microglia coculture reduced VEGFA expression in PTEN overexpressing (PTEN CoMG) compared to control cells (CTL CoMG). Student's *t-test* was used to analyze statistical significance. *p < 0.05.

Organotypic brain slice co-cultures showed a close interaction betwen glial species and tumor cells, indicating that a crosstalk between host and tumor cells takes place. To confirm the above described results and to investigate the role of PTEN in this process, we performed a Proteome Profiler™ Antibody Array. Supernatants from 48 h co-cultures of astrocytes or microglia with 231BR/CTL or 231BR/PTEN were incubated on antibody-spotted membranes of the Human XL Cytokine Array. Several cytokines were found to be differentially expressed between astrocyte co-cultures with 231BR/CTL and 231BR/PTEN (Figure 7A and Supplementary Figure S2). Aggrecan (ACAN) was the only factor with increased expression in co-cultures of astrocyte and 231BR/PTEN cells compared to 231BR/CTL co-cultures, whereas expression of angiopoietin 2 (ANGPT2), brain-derived neurotopic factor (BDNF), growth differentiation factor 15 (GDF15), EGF, cluster of differentiation 14 (CD14), interleukin 17A (IL17A), colony-stimulating factor 2 (CSF2) and basigin (BSG) was decreased in this setting. Interestingly, fewer differentially expressed factors were found in microglia co-cultures, (Figure 7B, and Supplementary Figure S2) which may explain the the stronger effects on astrocyte protrusion compared to the microglia migration observed in the organotypic co-culture. Expression of IL17A was decreased in 231BR/PTEN co-culture with microglia in a similar manner as with astrocytes. Additionally, vascular growth factor (VEGF), insulin-like growth factor-binding protein 2 (IGFBP2), and thrombospondin 1 (THSB1) expression was also reduced when microglia were co-cultured with PTEN overexpressing cells (Figure 7B). In summary, 231BR/PTEN cells showed reduced secretion of specific cytokine when co-cultured with either astrocytes or microglia.

To verify these results, we performed qPCR analyses and found only GM-CSF (CSF2) and EGF expression to be reduced in PTEN overexpressing cells (231BR/PTEN) compared to control cells (231BR/CTL) when co-cultured with astrocytes (Figure 7C all *p* < 0.05). Interestingly, the receptor for CSF2, CSF2RA, was not expressed on astrocytes in single culture but was significantly up-regulated when co-cultured with tumor cells. Activation of CSF2RA expression was PTEN-dependent (Figure 7C p < 0.05). When tumor cells were co-cultured with microglia, VEGFA secretion was reduced in 231BR/PTEN cells compared to 231BR/CTL cells (Figure 7C, p < 0.05).

## DISCUSSION

Distant metastasis formation is a multistep process, usually referred to as the metastatic cascade, including complex interactions between resident and tumor cells at all stages of the cascade [21]. Animal studies have shown that only a very minute percentage of tumor cells are capable of undergoing all these steps successfully and several studies have shown that outgrowth of tumor cells at distant sites is the most likely rate-limiting step in metastasis [22, 23]. Here, we describe the vital role of the tumor suppressor gene PTEN in the crosstalk between residential brain cells and tumor cells, which leads to rapid disease progression among patients with loss of PTEN.

In previous studies, we showed that EGFR- and HER2-driven pathways are frequently altered in brain metastases of breast cancer patients [12]. Here, we analyzed PTEN expression in a larger study population of brain metastases and showed that loss of PTEN is significantly associated with triple-negative breast cancer subtype and a shorter survival time after brain metastases resection. Recently Zhang et al., [24] described that PTEN expression in brain metastases can be specifically downregulated by specific microRNAs from astrocytes. This adaptive PTEN loss enhanced to outgrowth of the brain metastatic tumor cells. In order to address the functional role of PTEN in brain metastases formation and interaction with the brain microenvironment further, we overexpressed PTEN in the triple-negative breast cancer cell line MDA-MB-231 BR. The BR cell line was established from the parental MDA-MD-231 cell line following serial *in vivo* passaging to the brain [25]. We observed an increased activation of the AKT pathway in this brain seeking MDA-MB231BR subline. The AKT family consists of three members, Akt1, Akt2 and Akt3, which are closely related and highly conserved. Several studies have shown that the different functional isoforms have specific roles in regulating proliferation, apoptosis and migration in human cancer cells [26–28]. Interestingly, we identified Akt1 as the main activated AKT isoform in MDA-MB231BR cells. As expected, overexpression of PTEN in the MDA-MB-231 BR cells reduced AKT pathway activation, which was due to reduced Akt1 activity. Downregulation of Akt1 has been associated with reduced proliferation in a variety of cancer cells including cholangiocarcinoma cells [29], suggesting an important role of Akt1 in cancer cell proliferation. Our results suggest a specific role of Akt1 activation in breast cancer brain metastases due to a loss of PTEN.

Recent studies documented an intensive reciprocal crosstalk between tumor cells and resident brain cells that principally determines the survival of a tumor cell and it's outgrowth as a macrometastasis in the brain [4, 8, 30]. Our results also showed, irrespective of the negative astrocyte effect on cancer cell proliferation, interaction of glial cells with tumor cells induces a more aggressive phenotype, as indicated by increased migration and invasion. Interestingly, tumor cell proliferation was also inhibited when grown in CM from microglia treated with IFNγ and TNFα, whereas CM from microglia treated with TGFβ1 had no effect on tumor cell proliferation. As microglia treated by IFNγ and TNFα supposedly represent a population of M1 activated microglia and TGFβ1 treated cells a subpopulation of M2 activated microglia, the tumor cells in our experimental system seem to shift microglia towards a more M2-like phenotype [31]. The inhibitory effect on proliferation was more pronounced in PTEN overexpressing cells when co-cultured with astrocytes whereas no such effect was seen for microglia.

Both glial cells can highly activate MDA-MB-231 BR tumor cell migration in a similar manner as EGF stimulation. Both astro- and microglia similarly activated parental BR tumor cells, but migration of tumor cells was almost completely abolished when PTEN was overexpressed. The *ex vivo* organotypic brain experiments showed similar results. PTEN overexpressing cells possessed a significantly reduced capacity for invasion compared to the parental cell line. Furthermore, whereas both astro- as well as microglia interacted directly with the tumor cells in the 3D tumor plug, PTEN overexpression induced a lesser degree of astrocyte activation as indicated by the reduced length of the astrocytes in comparison to the parental tumor cell line. Interestingly, and more recently, Osswald et al. (2015) showed that many tumor cells in astrocytomas extend ultra-long membrane protrusions using these tumor microtubes as routes for brain invasion [20]. Gap43 and connexin 43 expression was shown to be important for the microtube formation, and to drive tumour cell invasion and proliferation [20]. Also in lung and breast cancer brain metastases, carcinoma-astrocyte gap junctions composed of connexin 43 were recently shown to promote brain metastasis formation [32]. Interestingly, in our study, both astrocytes as well as tumor cells upregulated the Gap43 expression when co-cultured together. Gap43 expression was, however, not dependent on PTEN expression. In line with this, we demonstrated that the astrocyte protrusions could also serve as guidance structures for the invading carcinoma cells if they fail to induce apoptosis in the cancer cells. This guidance could be controlled by a direct crosstalk between the astrocytes and cancer cells.

In regard to our previous results and the description of an intense PTEN-dependent crosstalk between tumor and astrocytes [24], we decided to investigate which cytokines and chemokines are capable of mediating these processes. Interestingly, astrocytes seemed to induce a larger PTEN-dependent chemokine activation than microglia. This could partially depend on the nature of the glial cells used. Whereas the astrocytes were primary cells, the microglia cell line is an SV40 large T antigen immortalized embryonic cell line and thus may not be activated as primary adult cells [33]. Among the PTEN-regulated chemo- and cytokines we verified differential expression of CSF2 (also known as GM-CSF), EGF and VEGFA by tumor cells. EGF is well-known for its ability to enhance the invasion of tumor cells, while CSF1/2 are able to recruit stromal cells, such as macrophages, to the tumor cells [34]. We found elevated levels of CSF2 expression in parental BR cell lines compared to PTEN-overexpressing cells. This overexpression lead to activation of the CSF2RA receptor in astrocytes when tumor cells were co-cultured with astrocytes. Furthermore, activation of the receptor was shown to be dependent on PTEN expression in tumor cells. GM-CSF is a cytokine that was originally described as a factor stimulating growth and differentiation of hematopoietic precursor cells. However, GM-CSF expression has also lately been detected on tumor cells and high levels of GM-CSF has been associated with poor survival in breast cancer [35]. Remarkably, in glioma cells knockdown of GM-CSF reduced microglia-dependent invasion and intracranial growth of glioma cells [36]. Moreover, GM-CSF has also been shown to activate the PI3K pathway [37]. Interestingly, whole genome analysis of brain metastasis of different tumor types demonstrated a significant gain of PI3K mutations in brain metastasis compared to the primary tumors [38], suggesting an important role for active PI3K/AKT signaling in adaption of tumor cells to the brain microenvironment. Thus, GM-CSF expression has not only an autocrine function in tumor cells but also important paracrine functions that can be counteracted by PTEN. GM-CSF seems to trigger or drive activation of tumor-associated astrocytes, supporting tumor growth, migration and invasion of breast cancer cells when the PI3K pathway is activated. Of course, other factors such as VEGF and EGF also play an important role in these processes.

In conclusion, several studies have shown the importance of crosstalk between residential brain cells such as glial cells and tumor cells [8]. Here, we describe the vital role of the tumor suppressor gene PTEN in these processes. In clinical BCBM specimens, we showed that loss of PTEN is significantly associated with both a triple-negative subtype and worse prognosis. Furthermore, upregulation of PTEN in a triple-negative breast cancer cell line lead to reduced migration and invasion to the brain, which was highly dependent on the crosstalk between tumor and glial cells, mediated by autocrine and paracrine activation of GM-CSF/ CSF2RA and AKT/ PTEN pathway on both astrocytes and tumor cells.

## MATERIALS AND METHODS

### Patient material

A tissue microarrays (TMAs) comprising of *n* = 131 formalin-fixed and paraffin-embedded (FFPE) BCBM tissue samples was processed as described by Harter et al. [39]. All tumor specimens were collected at the Institute of Neuropathology, University Medical Center Hamburg Eppendorf, Germany. Approval for this study was obtained from the local ethical committee (project number: MC-267/13) and have been performed in accordance with the ethical standards laid down in an appropriate version of the 1964 Declaration of Helsinki. Altogether 111 samples were analyzable for PTEN and follow-up survival data of 45 patients (survival time after tumor resection) was available.

### Immunohistochemistry

Immunohistochemistry for PTEN was performed using 4 μm thick slides and standard protocols as described earlier [12]. For immunohistochemical staining of PTEN, antigen retrieval was performed in sodium citrate buffer (Biogenex, Hamburg, Germany) followed by incubation with a PTEN antibody overnight at 4°C (1:50, 138G6; Cell Signaling Technology, Danvers, MA) and Dako REAL Detection System (Dako). Cytoplasmic staining was scored as negative expression (0), low expression (1), or high expression (2). For statistical analyses, expression was defined as either negative (0) or positive (1 and 2).

### Cell lines and cultures

HEK293T, MDA-MB-231, MDA-MB-468 and CHME3 cells were obtained from the ATCC (Manassas, USA), the brain metastatic MDA-MB-231 BR cell line was a kind gift from Dr. Takara (University of Texas) and the MDA-MB-231 SA cell line was provided by Dr. Guise (Indiana University School of Medicine) and primary human astrocytes were obtained from Thermo Fisher Scientific (N7805.100, Germany). The MDA-MB-231 SA and BR cell lines are sub-clonal cell lines obtained by serial passaging of the parental MDA-MB-231 cells in mice [25, 40]. The microglia cell line CHME3 is an SV40 large T antigen immortalized human embryonic cell line [33]. The MDA-MB-231 cell lines were authenticated using Multiplex Cell Authentication (Multiplexion, Heidelberg, Germany). Cells were grown in Dulbecco's modified Eagle's medium (DMEM). Human microglia cell line CHME3 was cultured in Minimal Essential Medium [33]. Each media was supplemented with 10% fetal bovine serum. Lentiviral particles were generated by co-transfection of expression vectors with psPAX2 packaging and pMD2.G envelope plasmids into HEK293T cells using TurboFect (Life Technologies, Eggenstein, Germany). Zsgreen-positive MDA-MB-231 BR control (231BR/CTL) and PTEN overexpressing (231BR/PTEN) cells were generated by lentiviral transduction with either control LeGO-SWITCH vector piZs2puro^++^tTR^+^ or overexpression vector pPTENiZs2puro^++^tTR^+^ (manuscript in preparation) and cultured in DMEM supplemented with 1 μg/ml doxycycline. Stable transfectants were selected in DMEM supplemented with 4 μg/ml puromycin. Suitable concentrations of doxycycline as well as puromycin were determined by titration.

For quantitative realtime-PCR (qPCR) and western blot analysis, tumor cells and astrocytes or microglia cells were cultured in a Boyden chamber (Thermo Fisher Scientific) separated by a transwell membrane with 0.4 μm pore size. For analysis of tumor cells 1.5 × 10^4^ 231 BR/CTL or 231BR/PTEN cells were seeded in 6-well plates and cultured for 24 h. Subsequently, 1.5 × 10^4^ astrocytes or microglia were seeded in inserted transwell inserts. Before collection of samples, cells were incubated for 48 h under standard conditions. For analysis of astrocytes and microglia, experiments were performed identically, except for culturing astrocytes or microglia in culture plates and tumor cells in the inserts.

For cytokine array analysis, conditioned media (CM) were produced by culturing astrocytes or microglia alone (CM_A_, CM_MG_), 10:1 with 231BR/CTL (CM_A+CTL_, CM_MG+CTL_) or 231BR/PTEN cells (CM_A + PTEN_, CM_MG+PTEN_). For proliferation assay analysis, astrocytes or microglia were additionally treated with 20 nM TNFα and 20 nM IFNγ (CM_A treat_, CM_MG M1_) or 20 nM TGFβ1 for 72 h (CM_MG M2_, all supplements obtained from R&D Systems, Wiesbaden, Germany). Supernatants were collected and cell debris was removed by centrifugation for 5 min at 25 000 rpm. The supernatants were used for the subsequent proliferation and cytokine arrays analysis.

### Immunoprecipitation of AKT isoforms and *in vitro* kinase assay

AKT isoform specific immunoprecipitation and *in vitro* kinase assays were performed as previously described [27]. In brief, direct immunoprecipitation was carried out by coupling of each AKT isoform antibody (Akt1; Cell Signalling Technology, MA, USA, Akt2; Santa Cruz Biotechnology, CA, USA and Akt3; Millipore GmbH, Schwallbach, Germany) to protein G-sepharose. AKT isoforms were precipitated overnight from whole cell lysates and subsequently incubated with GSK3α/βfusion protein (Cell Signalling Technology, MA, USA) and ATP (Cell Signalling Technology, MA, USA). Phosphorylation of GSK3α/β at S9/21 (Cell Signalling Technology, MA, USA) was analyzed by Western blot technique.

### Proliferation and migration

Analysis of cellular proliferation was done by MTT assay. 1 × 10^3^ 231BR/CTL or 231BR/PTEN cells were seeded in quadruplicates in 96-well plates in CM 1:1 DMEM supplemented with 1 μg/ml doxycycline for 96 h. Medium was changed every second day. Relative cell proliferation was estimated spectrophotometric at E578nm (reference: E690nm) using a Tecan Infinite^®^ 200 PRO microplate reader and ratio of treated (CM_A+CTL_, CM_MG+CTL_, CM_A + PTEN_, CM_MG+PTEN_, CM_A treat_, CM_MG M1_, CM_MG M2_) compared to untreated (CM_A_, CM_MG_) cells was calculated. Assays were repeated twice.

Migration was measured in a Boyden chamber assay. 1 × 10^5^ astrocytes or microglia were seeded in 6-well plates. Wells containing DMEM and DMEM supplemented with 20 nM EGF served as controls. Following 72 h incubation, 1 × 10^5^ 231BR/CTL or 231BR/PTEN cells were seeded in transwell inserts with 8 μm pores size in serum-free DMEM. The amount of migrated cells was estimated after 20 h by light microscopic analysis of fixed cells stained with crystal violet. Assays were done in triplicates and repeated three times.

### Western blot and cytokine array

Whole-cell extracts from pure 231BR/CTL or 231BR/PTEN and cocultures were prepared by direct lysis and sonication of cells in 2% SDS sample buffer containing phosphatase and protease inhibitors. Cell extracts were separated in denaturing 10% polyacrylamide gels and blotted onto a nitrocellulose membrane. Detection of proteins was performed by incubation with specific antibodies: PTEN (clone 138G6), panAKT (clone 11E7), phospho-AKT (Ser473, clone D9E), phospho-p44/42 MAPK (Erk1/2, Thr202/Tyr204, clone 197G2) (all from Cell Signaling Technology, Danvers, MA, USA) and MAPK (Erk 1, clone K-23, Santa Cruz Biotechnologies, Santa Cruz, CA). HSC-70 (clone B-6, Santa Cruz Biotechnologies) served as loading control and the HRP-conjugated anti-mouse and anti-rabbit antibodies were used as secondary antibodies (DAKO, Hamburg, Germany). Protein expression was quantified using LAS3000 Imager and AIDA Image Analyzer v.3.44. Fuji software (Raytest, Straubenhardt, Germany).

Cytokine secretion was estimated from CM by Proteome Profiler™ Antibody Array (Human XL Cytokine Array Kit, ARY022, R&D Systems, Wiesbaden, Germany) according to manufacturer's instruction. Signal intensity was quantified using densitometric analysis by ImageJ software (National Institutes of Health, Bethesda, Maryland). After normalization to control spots ratio between PTEN overexpressing (231BR/PTEN) compared to control samples (231BR/CTL) was calculated for astrocyte as well as microglia cocultures, using a cut-off of 2-fold. Assays were done in duplicates.

### Organotypic brain slice coculture system

C57BL/6 mice were decapitated between postnatal day six and eight for the organotypic brain slice coculture experiments, performed with interface technique following the previously described protocol [19]. These organotypic brain slices were cocultured with 1 × 10^5^ 231BR/CTL or 231BR/PTEN cells, embedded in 85% extracellular matrix (ECM gel; R&D Systems, Wiesbaden, Germany) for 96 h. The experiments were repeated twice with at least six sections per sample from 9 mice. Invasion was estimated in 45 (231BR/CTL cells) and in 47 brain sections (231BR/PTEN cells). Here, the complete contact area between brain slice and tumor spheroid was evaluated for invading tumor cells.

### Immunofluorescence staining of astro- and microglia in the organotypic brain slice coculture

Double staining of astro- and microglia was performed using anti-GFAP for 36 h at 4°C and anti-mouse-TRITC antibodies (both from Sigma, Munich, Germany) for 1 h at room temperature, followed by fluorescence dye-conjugated ILB4-Alexa Fluor 647 (Invitrogen, Karlsruhe, Germany) for 1 h at room temperature and DAPI counterstaining as described previously [19]. Samples were mounted in DAKO fluorescent mounting medium (Dako, Glostrup, Denmark), coverslipped and analyzed with confocal laser scanning microscope (LSM 510, Zeiss, Göttingen, Germany). Degree of invasion was evaluated from 0–+++. Grade of tumor invasion was based on following scoring system (0 = no cells; + < 1/3; ++ = 1/3 - 2/3; +++ ≥ 2/3 invaded cells) relating to fraction of contact section being invaded.

### qPCR analyses

Tumor cells and microglia and astrocyte cocultures were separated by FACS sorting based on the Zsgreen expression of the tumor cells. 500 ng total RNA was isolated using the NucleoSpin^®^ RNA (Macherey Nagel, Düren, Germany) including a DNase I digestion and reverse transcribed using the First Strand cDNA Synthesis Kit (Thermo Fisher Scientific, Darmstadt, Germany). qPCR was performed in triplicates using Maxima SYBR Green qPCR Master Mix (Thermo Fisher Scientific) and CFX96 Touch^™^ Real-Time PCR Detection System (Bio-Rad Laboratories, München, Germany). Oligonucleotide sequences are shown in supplementary Table S1. Relative gene expression was calculated by ΔΔCT method using RPLP0 as housekeeping gene, normalizing to universal human reference (UHR) and given as fold-change (FC). The experiments were performed in triplicates.

### Statistical analysis

Statistical analyses for Immunohistochemical staining results were performed by IBM SPSS statistics (version 19; IBM, Armonk, NY). Correlation between PTEN expression and clinical data, were calculated with Fisher's exact test or χ^2^-test. Survival was calculated by Kaplan-Meier curves and differences between normal and altered samples were examined by log-rank test. Significance of the different groups of organotypic slice cocultures was analyzed with Kruskal-Wallis test and Student's *t-test*. *P*-values ≤ 0.05 were considered statistically significant. In the figures *represents *p* < 0.05; **p < 0.01; and ***p < 0.001.

## SUPPLEMENTARY MATERIALS FIGURES AND TABLES


