# Supplementary material for: PTEN mediates the cross talk between breast and glial cells in brain metastases leading to rapid disease progression
Source: Oncotarget. 2016 Dec 20;8(4):6155–68. doi: 10.18632/oncotarget.14047 (PMC5351620; doi:10.18632/oncotarget.14047)
Supplement: Supplementary file 1 [file oncotarget-08-6155-s001.pdf]

PTEN mediates the cross talk between breast and glial cells in brain metastases leading to rapid disease progression

Supplementary Materials

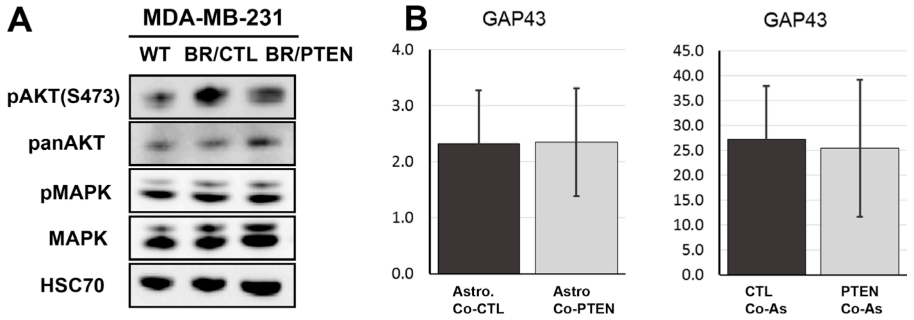

Supplementary Figure S1: (**A**) Immunoblot analysis of MAPK pathway protein expression in MDA-MB-231 (WT) cell line, brain-seeking (BR) sublines with either empty vector control (BR/CTL) or PTEN overexpression (BR/PTEN). HSC70 served as loading control (**B**) qRT-PCR results for GAP43.

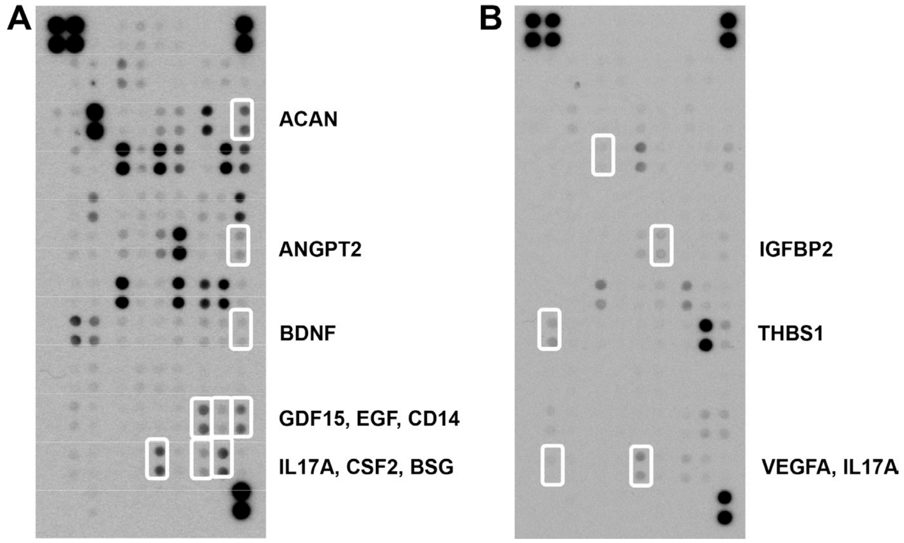

Supplementary Figure S2: Cytokine secretion in astrocytes and microglia.

Supplementary Table S1: qRT-PCR primers used in this study

Gene	mRNA ID	FW Primer (5' → 3')	RV Primer (5' → 3')	T(ann) °C	bp cDNA	Reference
ACAN	NM_001135.3	CACACCTGAGCAGCATCGT	GGTAGTCTTGGGCATTGTTGTT	60	67	own primer
ANGPT2	NM_001147.2	TGAACCAAACAGCGGAGCAA	CTCGTGGTCTGATTTAATACTTGGG	58	70	own primer
BDNF	NM_170735.5	GTAACGGCGGCAGACAAAAA	GTAGTTCGGCACTGGGAGTT	58	191	own primer
BSG1-2	NM_198589.2	GCGAGGAATAGGAATCATGG	TCTACGGTAGTGAAGACTGTG	60	107	Menck et al., 2015
CD14	NM_000591.3	ATAACCTGACACTGGACGGG	GGAGTTCATTGAGCCCTCGT	60	74	own primer
CSF2	NM_000758.3	ACACTGCTGCTGAGATGAATG	CTGGAGGTCAAACATTTCTGAGATG	58	59	own primer
CSF2RA	NM_172249	ACCCTGTACGTGCTTCCTTCG	CCACTGTTCGCAGATCCGATT	58	146	own primer
EGF	NM_001963.4	TGGGTATGACCTACAACTG	GCATGTGTCGAATATCTTGAG	58	94	Menck et al., 2015
GAP43	NM_001130064.1	GAGCAGCCAAGCTGAAGAGA	CTTGGTCAGCCTCAGGTTCC	60	104	own primer
GDF15	NM_004864.2	ATACTCACGCCAGAAGTGC	CCCGAGAGATACGCAGGTG	60	61	own primer
IGFBP2	NM_000597.2	GCCCTCTGGAGCACCTCTACT	CATCTTGCACTGTTTGAGGTTGTAC	60	80	own primer
THBS1	NM_003246.2	GATGCCTGCTTCAACCACAA	GGCTTGCACACCTGTTTGTT	58	149	own primer
VEGFA	NM_001287044.1	CTGAGGAGTCCAACATCAC	GTCTTGCTCTATCTTTCTTTGG	58	125	Menck et al., 2015

Menck K et al., Tumor-derived microvesicles mediate human breast cancer invasion through differentially glycosylated EMMPRIN. J Mol Cell Biol. 2015 Apr;7(2)
